# High-performance visible-enhanced SnS/Si photodetectors fabricated by laser ablation: effect of laser fluence on optoelectronic properties

**DOI:** 10.1039/d6ra05741a

**Published:** 2026-07-31

**Authors:** Fatima M. Abdulaziz, Raid A. Ismail, Alwan M. Alwan

**Affiliations:** a College of Applied Science, University of Technology Baghdad Iraq raidismail@yahoo.com

## Abstract

Tin(ii) sulfide (SnS) is one of the most promising two-dimensional (2D) semiconductors for optoelectronic applications. SnS nanostructures have received substantial attention in the research field because of their earth-abundant nature, low production cost, and environmentally friendly composition, making them highly suitable for photovoltaic and photocatalytic applications. In this study, tin sulfide nanoparticles were prepared *via* laser ablation of a Sn target in an aqueous Na_2_S solution under different laser fluences of 532 nm pulses of a Nd:YAG laser. X-ray diffraction (XRD) results confirmed that the synthesized SnS nanoparticles are polycrystalline in nature with an orthorhombic α-SnS phase that shows a preferred orientation along the (110) plane for all synthesized samples. Field-emission scanning electron microscope (FESEM) investigations indicate that the film prepared at 45 J cm^−2^ was compact and dense, with cauliflower-like agglomerated structures and is converted into partially overlapping quasi-spherical clusters resembling cauliflower when the laser fluence increases to 50 J cm^−2^. At 60 J cm^−2^, nanorod-like morphology was observed. Raman spectroscopy reveals that the synthesized SnS exhibits vibrational modes of Sn–S bonds. The optical characteristics demonstrated that the optical energy gaps of SnS prepared at 45, 50, and 60 J cm^−2^ was 1.62, 1.49, and 1.72 eV, respectively. The p-SnS/n-Si heterojunction fabricated at a laser fluence of 50 J cm^−2^ exhibits the best rectifying properties and maximum photocurrent. The maximum responsivity, detectivity, and external quantum efficiency was 1.45 A W^−1^, 4.9 × 10^11^ Jones, and 399%, respectively, at 450 nm for the SnS/n-Si photodetector prepared at 50 J cm^−2^. The transient current–time photoresponse of the photodetector was investigated as a function of laser fluence. This work shows that controlling the laser fluence in the PLAL process using a PVA-Na_2_S medium is an efficient strategy to tailor the structural and optoelectronic properties of SnS nanoparticles, thereby improving the performance of SnS/Si photodetectors through a simple and eco-friendly synthesis route.

## Abstract 2

Tin(ii) sulfide (SnS) is one of the most promising two-dimensional (2D) semiconductors for optoelectronic applications.

## Graphical abstract

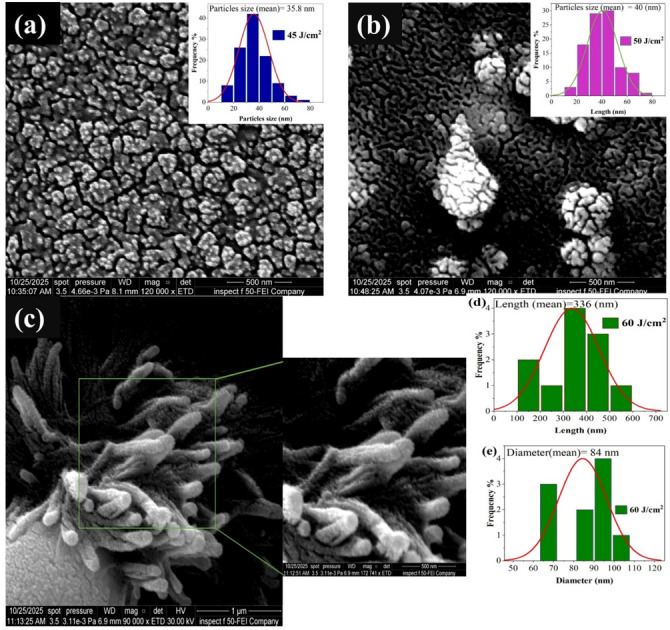

## 1 Introduction

In recent years, significant advancements have been made in nanotechnology, with low-dimensional nanostructures attracting considerable scientific interest for their high-performance and rapid-response photodetectors and electronic devices. They can be applied in optoelectronic applications and can be detected in the UV (250–400 nm), visible (450–800 nm) or near-infrared (900–1700 nm) region.^1,2^ Photodetectors are used in device fabrication and detection to convert the incident light signal into an electrical signal using the photoelectric effect in semiconductor materials. They have been extensively used in optical communications, biomedical sensing, and photoelectric imaging.^3,4^ Recent reports on optoelectronic materials reveal that nanomaterials and nanocomposites prepared by solution-processable methods can serve as active layers in the fabrication of large-area photodetectors that are non-toxic and of low cost.^5–7^ The most commonly reported photodetectors are focused on inorganic semiconductor materials, in particular GaN, Si, and InGaAs.^8^ To facilitate the practical implementation of nanoscale photodetectors, it is essential to identify new materials that can be readily manipulated in size, shape, and composition, and that can be integrated with current devices. In this context, tin sulfide (SnS) has been of great interest due to its direct band gap (∼1.3 eV), which is between that of silicon (Si) and gallium arsenide (GaAs), and also because of its potential as a material for photostimulation and visible-light-driven supercapacitors.^3,9,10^ In addition, SnS has a high absorption coefficient (>10^4^ cm^−1^) in the visible region, Hall mobility up to 100 cm^2^ V^−1^ s^−1^, and tunable carrier densities in the range of 10^15^–10^18^ cm^−3^.^11^ SnS crystallizes at room temperature in an orthorhombic double-layered low-symmetry structure with lattice parameters *a* = 0.4329 nm, *b* = 1.1192 nm, and *c* = 0.3984 nm. It is a low-cost material, non-toxic, has high moisture stability, and high electrical conductivity, which are all desirable properties for solar cell applications. SnS has a number of binary phases SnS, Sn_2_S_3_, Sn_3_S_4_, Sn_4_S_5_ and SnS_2_ depending on the Sn/S ratio.^12^

Various techniques have been used to prepare SnS nanoparticles, such as hydrothermal/solvent synthesis, plasma synthesis, electrodialysis, microwave synthesis, high-temperature self-diffusion synthesis, and precipitation.^12–15^ However, all of these techniques require harsh conditions, such as high temperatures and pressures, and the use of toxic chemicals. Recent reported data have indicated that laser ablation produces SnS nanoparticles with diverse morphological structures, such as nanoblooms, nanorods, nanospheres, nanosheets, nanocrystals, nanowires, and others, to study their optical and electrical properties.^16^ This method has several advantages over other conventional methods, including its simplicity, high purity, ease of controlling its structure and shape, and suitability as an environmentally friendly synthesis method. However, its ability to operate in different environments and the potential to access new crystalline phases and sizes.^17^

In contrast to other conventional techniques for the preparation of nanoparticles by pulsed laser ablation, which employ pre-synthesized SnS targets or sulfur-containing organic solvents, the present work adopts a different synthesis strategy by ablating a pure metallic Sn target in a mixed PVA–Na_2_S solution. In this approach, Na_2_S serves as the sulfur source for the *in situ* formation of SnS nanoparticles, while PVA acts as a stabilizing agent to suppress nanoparticle agglomeration during synthesis. Additionally, the proposed approach is more cost-effective because metallic Sn targets are less expensive than SnS targets. Moreover, the influence of laser fluence on the structural evolution, optical properties, and photodetection performance of p-SnS/n-Si heterojunctions is systematically investigated. This integrated approach offers an eco-friendly and cost-effective route for tailoring SnS nanostructures and optimizing their optoelectronic performance.

Various laser methods like pulsed laser deposition, laser irradiation, laser ablation *etc.* have been used for comprehensive studies of SnS nanoparticles synthesis. Kadhim *et al.*^18^ reported the fabrication of nanocrystalline SnS thin films using pulsed laser deposition, where tin sulfide (SnS) nanoparticles were synthesized *via* irradiating compressed SnS powder target with a Nd:YAG laser (700 mJ) with different laser pulses of (200–350 pulses) and a frequency of the laser (10 Hz). According to Johny *et al.*^19^ SnS nanoparticles were prepared using pulsed laser ablation in liquid (PLAL), where Nd:YAG laser pulses of 532 nm wavelength were used to ablate the SnS target. Mostafa *et al.*^20^ used a Nd:YAG laser to synthesis high-purity SnS nanoparticles in DMSO as a sulfur source. Hwang *et al.*^21^ demonstrated Synthesis of SnS nanoparticles using the PLAL by irradiating of Sn in dimethyl sulfoxide (DMSO) as the sulfur source. Sreekala *et al.*^22^ introduced hybrid SnS–graphene thin films as a broadband photodetector. The fabrication involved irradiating SnS powder with a 532 nm Nd:YAG laser to prepare SnS nanocolloids. The obtained nanocolloids were then mixed with graphene. To deposit SnS–graphene hybrid films using spin-coating and subsequent vacuum annealing treatments. Averchenko *et al.*^23^ fabricated a broadband and fast-switching SnS photodetector with a photosensitivity of 4 A W^−1^ by preparing centimeter-scale tin sulfide tracks from a single-source precursor of tin(ii) *o*-ethylxanthate using the CW laser technique at 532 nm.

In this context, we report the synthesis of tin sulfide (SnS) nanoparticles *via* laser ablation of a tin target in a mixed solution of polyvinyl alcohol (PVA) and Na_2_S. Subsequently, the effects of laser fluence on the optical, electrical, and structural properties of the synthesized SnS nanoparticles were investigated and discussed. To fabricate a high-performance SnS/Si photodetector, laser fluence was systematically optimized. The method employed in this work represents an environmentally friendly, cost-effective, and sustainable approach for the synthesis of high-purity tin sulfide nanoparticles with tunable physical and electrical properties.

## 2 Experimental

**Fig. 1:**
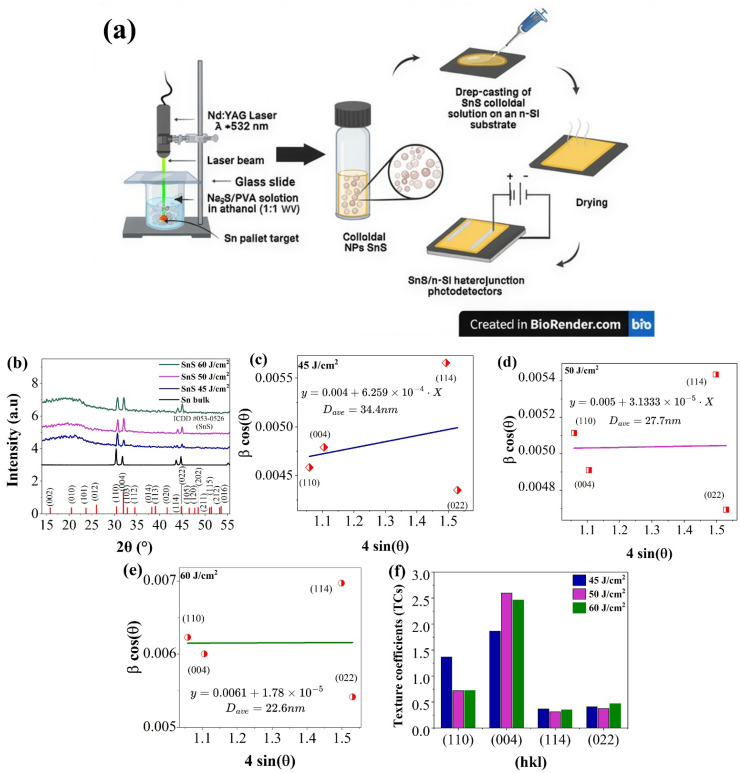
(a) Experimental steps of preparation of SnS nanoparticles and SnS/Si photodetector, (b) X-ray diffraction (XRD) patterns of SnS nanoparticles, (c–e) Williamson–Hall plots for samples prepared at laser fluences of 45, 50, and 60 J cm^−2^, respectively, and (f) texture coefficient histograms of SnS films at different laser fluences.

In this study, tin sulfide nanostructures were synthesized using a one-step laser ablation process in liquid. This technique involves irradiation of high-purity Sn pellets supplied with laser pulses, placed at the bottom of a glass vessel containing 2.5 mL of an aqueous sodium sulfide (Na_2_S flakes purchased from CHD – Central Drug House, India) solution. A 2.5 mM Na_2_S solution was prepared (used as the sulfur source) by dissolving 12 mg of sodium sulfide in distilled water. Subsequently, 0.1 g of PVA was added to the solution and mixed with ethanol in a 1 : 1 volume ratio, total volume 50 mL with continuous stirring for 10 min to ensure complete homogenization. PVA was introduced to enhance dispersion of colloidal nanoparticles and to reduce the tendency of the nanoparticles to aggregate, a low PVA content of 0.22 wt% was used to obtain good colloidal stability without significantly affecting the synthesis process. A Q-switched Nd:YAG laser (*λ* = 532 nm, second harmonic) was used with the beam radius of 1 mm, the laser energy of 400, 450 and 550 mJ, the pulse duration of 7 ns, the pulse repetition frequency of 3 Hz, and the laser fluences of 45, 50 and 60 J cm^−2^, corresponding to approximately 500 laser pulses. High-purity tin (99.9%) was compressed, and tin powder was compressed using a hydraulically driven compressor at 6 tons to make tin pellets with the thickness of 2 mm and the diameter of 10 mm. Fig. 1(a) shows the schematic diagram of laser ablation of SnS NPs in a liquid system. A convex converging lens with an 8 cm focal length was used to focus the laser beam on the Sn target. The glass vessel was covered with a thin glass slide to prevent the vapor from reaching the laser-focused lens and attenuating the laser energy. The ablation time was adjusted to be ∼167 s for all samples.

The surface morphology of the SnS nanoparticles was studied using a FESEM (FEI Company, INSPECT F50) field emission scanning electron microscope. The structural properties of SnS nanoparticles were examined using an X-ray diffractometer (XRD-6000, Shimadzu, Japan) using a CuKα source. Photoluminescence (PL) spectra were obtained using a spectrophotometer (FluoroMate FS-2 Spectrometer). The optical absorbance of the colloidal SnS nanoparticles was measured by using a UV-Vis double-beam spectrophotometer (Lambda 750, PerkinElmer). Raman spectroscopy was conducted on colloidal SnS nanoparticles within an aqueous-ethanol mixture utilizing a Raman spectrometer (Bruker Optics). Fabrication of SnS/Si photodetector was accomplished by deposition of a layer of SnS nanostructure on the silicon substrate, as shown in Fig. 1(a). The silicon used in this work was single crystal n-type with Si (111) orientation having an electrical resistivity of 10 Ω cm and with 1 cm^2^ area. The silicon substrates were cleaned using RCA cleaning method. To guarantee consistent coverage of the 1 cm^2^ active area, approximately twenty drops of the prepared colloidal solution were applied under the same circumstances. The deposited SnS film had an average thickness of approximately 515 nm, as determined from cross-sectional FESEM analysis. There was no post-deposition annealing; all depositions were carried out at room temperature. An ECOPIA HMS-3000 Hall measurement system in the Van der Pauw configuration was used to perform Hall-effect measurements. Al film and silver paste were used to create four ohmic contacts for SnS films that were deposited on glass substrates with a thickness of about 14 µm. Using an interdigitated mask to act as ohmic contacts, aluminum (Al) film was deposited onto the SnS layer and the back of the silicon substrate using the thermal evaporation technique to prepare the photodetector; the electrodes were separated by 1 mm, as seen in Fig. 1(a). The current–voltage characteristics and responsivity of SnS NPs/n-Si photodetectors at dark and illumination conditions were measured at room temperature using a regulated DC digital power supply, an electrometer (UNI-T UT33C), calibrated Jobin-Yvon monochromator, and a halogen lamp. Current time photoresponse of the photodetector was investigated to estimate the rise and fall times of the photodetectors.

## 3 Results and discussion

The formation mechanism of SnS nanostructures during laser ablation in liquid (LAL) can be explained based on the sulfurization of laser-ablated tin species as follows: when high-power laser pulses irradiate the Sn target immersed in Na_2_S solution, tin ions and atoms are ejected into the surrounding Na_2_S medium, forming a plasma plume. The plasma expands, Na_2_S dissociates in the aqueous medium to provide S^2−^ ions, which react with the ablated Sn atoms and ions to form SnS nanostructures, according to the following chemical reaction:
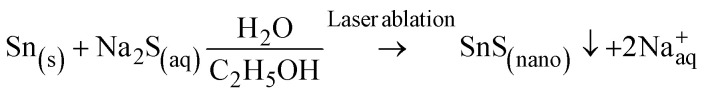


This product precipitates in the solution as colloidal nanoparticles or other morphologies. After SnS formation, the color of the solution changed from transparent to dark brown, which depends on the laser fluence, as shown in photographs of SnS colloids prepared at various laser fluences. The XRD pattern of the SnS NPs synthesized at various laser fluences is illustrated in Fig. 1(b). Four peaks were observed at 2*θ* = 30.6°, 32.06°, 43.8°, and 44.9° in the XRD pattern of SnS synthesized at a laser fluence of 45 J cm^−2^ corresponding to (110), (004), (114), and (022) planes, respectively. All observed XRD peaks belong to the orthorhombic SnS phase (ICDD #053-0526). The diffraction peak corresponding to the (110) plane exhibited the highest intensity, indicating a preferred orientation along this plane. This preferential orientation can be attributed to the growth kinetics and morphology of the nanoparticles formed during laser ablation. A slight shift of 2*θ* of the reflection peaks was observed at higher laser fluences between 50 and 60 J cm^−2^. This could be indicative of the presence of internal lattice strain caused by the rapid formation and quenching of nanoparticles at high laser fluence as suggested by the variation of the XRD peak profiles. No Sn related XRD peaks were observed indicating the complete reaction of Sn with sulphur species to form SnS.

The lattice strain (*ε*) in the SnS nanoparticles was determined from X-ray diffraction (XRD) analysis1
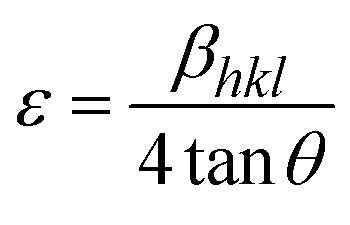
where *β*_*hkl*_ is the XRD peak's full width at half maximum (FWHM).

The following formula can be used to calculate the dislocation density (*δ*) in SnS nanoparticles2
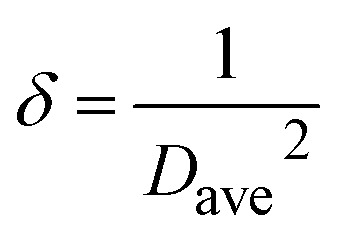
where *D*_ave_ is the SnS film's average crystallite size.

The average crystallite size (*D*) of SnS nanoparticles prepared at various laser fluences was calculated using Scherrer's formula:3
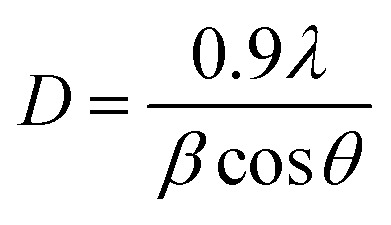
where *λ* is the CuKα source's X-ray wavelength (0.1549 nm) and *β* is the full width at half maximum in radians. Table 1 presents the crystallite size of SnS NPs prepared at various laser fluences.

**Table 1:** XRD analysis for synthesized SnS nanoparticles

Laser fluence (J cm^−2^)	2*θ* (degree)	(*hkl*)	D–S and Wilson method			W–H plot		
			*D*_ave D–S_ (nm)	Strain *ɛ*_D–S_	Dislocation density, *δ* × 10^15^ (lines per m^2^)	*D*_W–H_ (nm)	*ɛ*_W–H_ × 10^−4^	*δ* × 10^15^ (lines per m^2^)
45	30.6	(110)	25.8	0.013	1.5	34.4	1.56	0.84
50	30.7	(110)	24.6	0.026	1.6	27.7	0.078	1.29
60	30.6	(110)	20.25	0.032	2.4	22.6	0.045	1.95

The lattice constants *a*, *b* and *c* of SnS films grown at laser fluences 45, 50 and 60 J cm^−2^ were calculated by using XRD analysis. Using the following equation:4
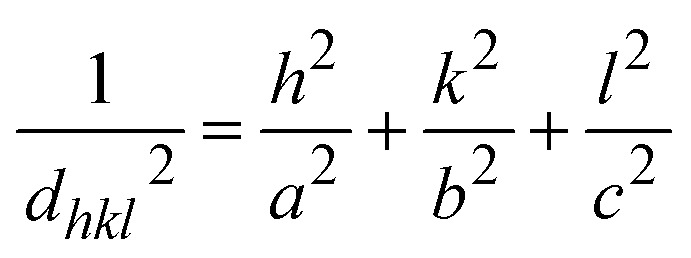
where *d*_*hkl*_ is the interplanar distance corresponding to the plane *hkl*. The values obtained were 3.98, 4.34 and 11.2 Å, respectively. These values are in good agreement with reported lattice constants for bulk SnS.^11^

The crystallite size, lattice strain, and dislocation density were evaluated using the Debye–Scherrer and Williamson–Hall methods. The Williamson–Hall analysis was performed according to eqn (5) using OriginPro 2024 software:^24^5
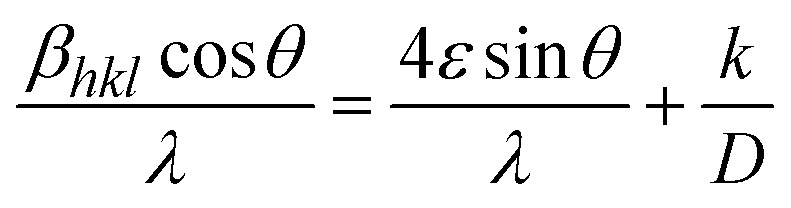
where *D* denotes the crystallite size, while *ε* represents the strain. As shown in Table 1, the structural parameters illustrated in Fig. 1(c–e) demonstrate evolutions of the SnS/PVA composite at laser fluences of 45, 50, and 60 J cm^−2^. An observation was made that increasing the laser fluence resulted in a decrease in crystallite size. This decrease in crystallite size is associated with a change in microstrain and dislocation density as evaluated using the Williamson–Hall method. This decrease can be attributed to the increased ablation efficiency at higher laser fluence, which promotes nucleation over crystal development and results in the synthesis of smaller nanoparticles. The rapid cooling and the increased collision frequency during the ablation process may induce lattice distortions, leading to increased microstrain. It was also observed that the dislocation density increased with increasing laser fluence due to enhanced lattice defect formation. The observed phenomenon corresponds to an increase in the number of internal defects that indicates a decrease of the crystalline quality.

The crystallite sizes obtained from the Williamson–Hall method were slightly different from those obtained from the Debye–Scherrer equation which may be due to microstrain effects in the SnS films. The incorporation of PVA may establish non-uniform internal strain due to its interaction with the surface of the nanoparticles, resulting in additional broadening peaks. Although higher laser fluence may promote particle collision and partial coalescence in the colloidal solution, the XRD results indicate that crystallite refinement dominates under the present experimental conditions. These structural changes are expected to play an important role in determining the subsequent morphological, optical, and optoelectronic properties of the synthesized SnS nanoparticles.

These structural changes are expected to influence the optical properties of the material and to permit tuning of the optical band gap with laser fluence. These results show that laser fluence plays a key role in controlling crystallite size, microstrain and dislocation density *via* interconnected structural changes.

Eqn (6) was used to analyze the crystal orientation preference along the crystal plane (*hkl*) of X-ray diffraction data from SnS samples. This equation, known as the Harris equation, was reformulated by Müller, Chernok, and Beck, and is as follows:^25^6
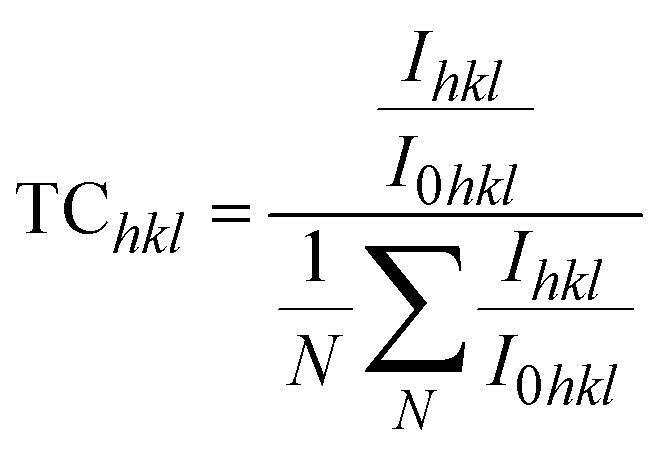
where *N* is the number of reflections studied, *I*_*hkl*_ is the X-ray diffraction intensity of the peak, and *I*_0*hkl*_ is the X-ray diffraction intensity of the non-crystalline reference material (ICDD #053-0526). The TC value is obtained by dividing the intensity of the observed sample by the intensity of the reference sample corresponding to the observed sample. The preferential orientation of a particular orientation is determined by calibration with respect to the average of all the reflection intensities. TC > 1 indicates preferred orientation. A randomly oriented structure will result in a TC_*hkl*_ value of 1 for a sample. The variation in the value of TC_*hkl*_ clearly shows that it is significantly influenced by laser fluence as reflected in the XRD patterns (Fig. 1(f)) and summarized in Table 2. The (110) plane remained the preferred orientation for all SnS samples. Although the corresponding TC value gradually decreased from 2.49 to 1.78 with increasing laser fluence, it remained above unity, indicating that preferential crystal growth along the (110) direction was maintained throughout the investigated laser fluences range. This behavior can be attributed to crystal rearrangement and the preservation of preferred growth along the (110) plane despite the increase in lattice defects and microstrain.^26,27^ Among the investigated fluences, the sample prepared at 50 J cm^−2^ exhibited the best balance between crystallite refinement and structural ordering, which is expected to contribute to its superior optical and optoelectronic performance, as discussed in the following section.

**Table 2:** Texture coefficient (TC) values of SnS–PVA nanoparticles prepared at different laser fluences are reported

Laser fluence (J cm^−2^)	2*θ* (°)	(*hkl*)	TC	Interpretation
45	30.6	(110)	2.49	Preferred
50	30.7	(110)	1.94	Preferred
60	30.6	(110)	1.78	Preferred

**Fig. 2:**
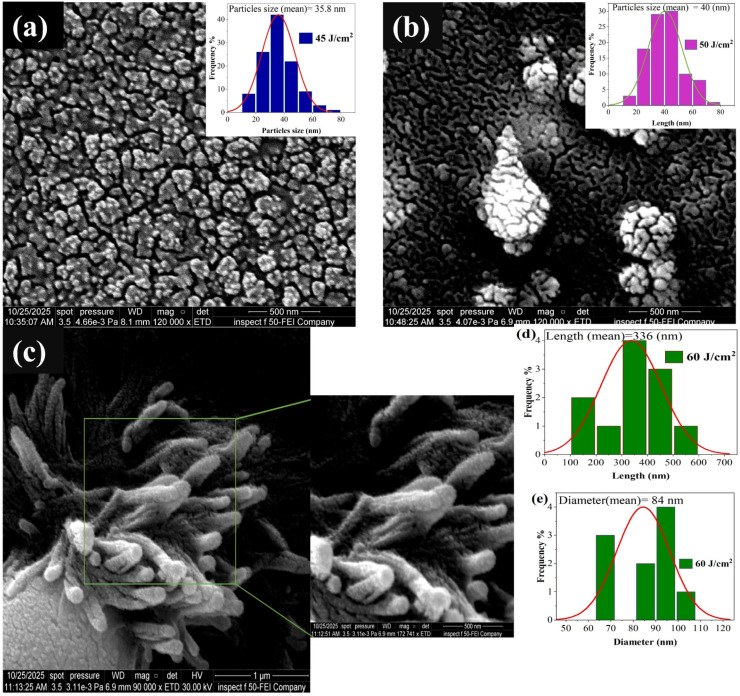
FESEM micrographs and particle size analysis of SnS nanoparticles prepared at various laser fluences: (a) FESEM image of SnS nanoparticles at 45 J cm^2^; inset is particle size distribution histogram for the corresponding sample; (b) FESEM image of SnS nanoparticles at 50 J cm^−2^: inset is particle size distribution histogram for the corresponding sample, (c) FESEM image of SnS nanorods sample prepared at 60 J cm^2^; (d) nanorod length distribution histogram, and (e) nanorod diameter size distribution histogram.

Fig. 2 shows a field-emission scanning electron microscope (FESEM) image of the SnS nanoparticles. As demonstrated in Fig. 2, the surface morphology of synthesized SnS nanoparticles was found to be strongly dependent on the laser fluence. The FESEM image in Fig. 2(a) shows a homogeneous, compact, cauliflower-like agglomerated structures formed *via* nucleation sites. The presence of PVA layers has a notable role in stabilizing these nuclei by steric hindrance, which prevents extreme agglomeration and favors a fine particle size distribution.^28^ The particle size distribution was determined using Image J software an average particle size of SnS nanoparticles was 35.5 nm. This indicates controlled growth at relatively low laser fluence.

At 50 J cm^−2^, the morphology evolved into partially overlapping quasi-spherical cauliflower-like clusters, as shown in Fig. 2(b). Also, the FESEM image confirms an increase in SnS particle size due to particle agglomeration. The resulting structure exhibited a porous heterogeneous morphology. This is due to the high temperature of ablated species and the formation of high-density plasma within the liquid, which increases collision rates and the probability of coalescing particles. Additionally, the polymeric environment allows the controlled diffusion of nanoparticles, which means that it is possible to partially fuse without the total collapse of the structure.^29^ The particle size determined from FESEM was larger than the crystallite size estimated from XRD because FESEM measures the overall particle dimensions, whereas XRD determines the size of coherent crystalline domains. Therefore, each particle may consist of several crystallites. Further increasing the laser fluence to 60 J cm^−2^, a remarkable morphological transformation of the particles was observed, where the particles turned into morphology that resembled nanoflowers or nanorod-like clusters. This may be ascribed to the high plasma temperature, high ejected particle density, and high vapor pressure. This leads to fast supersaturation of the ablated species and anisotropic growth. In this process, the PVA acts as a flexible template that not only prevents aggregation but also controls the aggregation process of complex-shaped particles.^30^ Statistical analysis (Fig. 2(d) and (e)) shows that the average nanorod length was ∼336 nm and the average diameter was ∼84 nm, thus confirming asymmetric growth and a high degree of structural evolution.

The laser fluence plays a key role in controlling the morphological evolution of SnS–PVA nanostructures, where the system gradually transitions from initial nucleation-dominated nanoparticle formation to the development of more complex hierarchical assemblies. This result has also been reported in chalcogenide–polymer nanocomposites prepared *via* laser-assisted methods, where the interplay between laser fluence and polymer-mediated stabilization strongly affects the structural arrangement.^31,32^

**Fig. 3:**
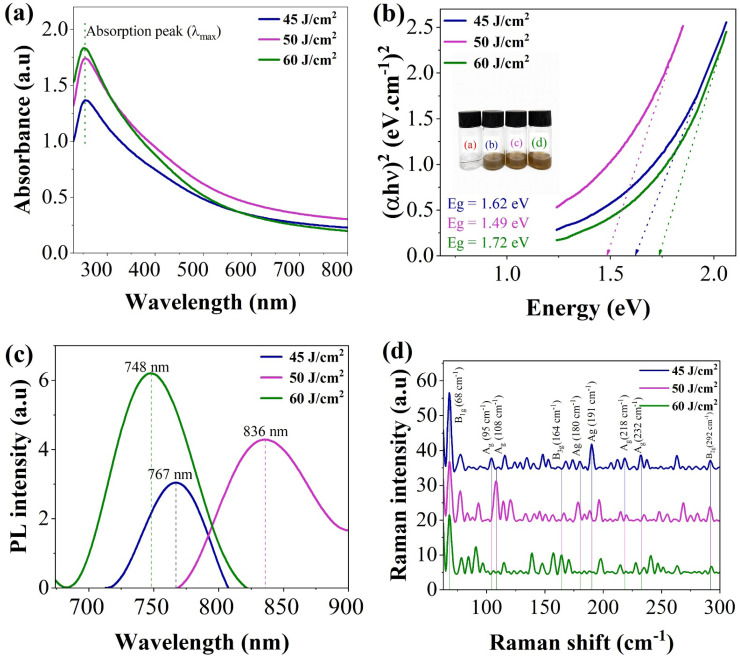
Optical properties of SnS–PVA nanostructures fabricated at different laser fluences: (a) optical absorption spectra of SnS nanoparticles, (b) optical band-gap estimation of SnS–PVA films, inset is photograph of freshly prepared colloidal SnS–PVA nanoparticles solution, (c) photoluminescence (PL) emission spectra of SnS–PVA films, and (d) Raman spectra of SnS nanoparticles synthesized at various laser fluences.

Fig. 3(a) displays the absorption spectra of colloidal SnS nanoparticles prepared at various laser fluences. The spectra exhibit shows the plot of a broad absorption band from 230 to 800 nm with prominent broad peaks at ∼256 nm for both the samples synthesizes with 45 and 50 J cm^−2^, and a noticeable blue shift of the absorption for the 60 J cm^−2^ sample. The sample prepared at a laser fluence of 50 J cm^−2^ exhibited the highest absorbance, which is consistent with the morphological evolution observed by FESEM, and contributed to its improved optical response, reflecting the structural and morphological changes induced by the applied laser fluence. Fig. 3(a) shows a shift in the absorption peak with respect to the increase in laser fluence. This change is attributed to the change of particle size, morphology, and electronic structure caused by variations in laser fluence.

Fig. 3(b) shows the plot of variation of (*αhν*)^2^ with photon energy (*hν*). The direct optical energy gap of SnS nanoparticles can be obtained by extrapolating the linear part to the photon energy axis, according to Tauc's equation:^33^

7 *αhν* = *A*(*hν* − *E*_g_)^*n*^

where *n* is the exponent of the type of electronic transition, *A* is a constant that depends on the type of material, and *α* is the absorption coefficient. For direct allowed and forbidden transitions, the values of *n* are 1/2 and 2, respectively, as illustrated in Fig. 3(b). The values of the direct energy gap of the SnS samples were estimated and found to be 1.62, 1.49, and 1.72 eV for the samples prepared at 45, 50, and 60 J cm^−2^, respectively. The optical band gap exhibited a non-linear dependence on laser fluence, decreasing from 1.62 eV at 45 J cm^−2^ to 1.49 eV at 50 J cm^−2^, followed by an increase to 1.72 eV at 60 J cm^−2^. The lower energy gap obtained at 50 J cm^−2^ is associated with the morphological evolution of the synthesized SnS nanoparticles. FESEM observations revealed the formation of partially overlapping cauliflower-like structures. This morphological evolution is consistent with the red shift of the absorption edge observed in the UV-Vis spectra and the corresponding reduction in the optical band gap.^34–36^ In addition, XRD analysis showed a gradual decrease in crystallite size together with an increase in lattice strain and dislocation density as listed in Table 1. These structural changes can modify the electronic structure of SnS, leading to a blue shift of the absorption edge and, consequently, a wider optical energy gap. Generally, the optical behavior of the synthesized SnS nanoparticles is mainly determined by the combined effects of morphology and crystal structure, which are strongly influenced by the applied laser fluence. In addition, the presence of PVA may have contributed to the stabilization of the nanoparticles during laser ablation, thereby improving their dispersion in the colloidal solution. This effect may indirectly influence the optical properties of the synthesized SnS nanoparticles, in agreement with previous reports by Alruwaili *et al.*^37^. The lower optical band gap obtained at 50 J cm^−2^ is expected to contribute to the enhanced photoresponse of the fabricated p-SnS/n-Si photodetector. A detailed discussion of the photodetector performance is presented in the following section.

Fig. 3(c) shows the effect of laser fluence on photoluminescence PL spectra of SnS nanostructures. PL investigations were performed at room temperature with an excitation wavelength of 532 nm. The synthesized SnS at 45 J cm^−2^ shows a PL emission peak at 767 nm corresponding to 1.61 eV. The presence of PVA has been shown to regulate the size and distribution of the nanoparticles, hence improving the optical properties of the SnS. This emission is due to radiative recombination of electron–hole pairs *via* defect states and localized trap levels in the band gap. The SnS nanoparticles synthesized at 50 J cm^−2^ exhibited a PL band at 836 nm, corresponding to 1.48 eV. PL emission peak centered at 748 nm (1.65 eV) was observed for the sample prepared at 60 J cm^−2^, which may be attributed to band–band recombination between free electrons and deep-level defect states within the band gap. Compared with the 50 J cm^−2^ sample, the emission peak of the 60 J cm^−2^ sample shifts toward shorter wavelengths, showing a blue shift consistent with the increase in the optical energy gap, as shown in Table 3. This finding can be explained by a partial improvement in crystallinity and structural reorganization at high laser fluence, although structural defects persist. This observed PL emission mainly belongs to defect-assisted radiative recombination involving sulfur vacancies and localized trap states within the SnS nanoparticles, indicating enhanced emission intensity at higher laser fluences.^38,39^ Furthermore, PVA is involved in the stabilization of SnS nanoparticles and affects their surface-related optical properties. This behavior can modify the PL intensity and peak position by varying the defect density and recombination processes.

**Table 3:** Comparison of the optical band gap and spectral shift behavior of SnS–PVA nanoparticles obtained from UV-visible and PL measurements

Laser fluence (J cm^−2^)	*E*_g_ UV-visible (eV)	*E*_g_ PL (eV)	Relative shift type
45	1.62	1.61	No obvious shift
50	1.49	1.48	Slight red shift
60	1.72	1.65	Red shift

This increase in the laser fluence may facilitate the existence of extra defect states in the band gap, which agrees with FESEM and the Raman analysis. Generally, PL behavior is largely governed by defect-related transitions.

These results indicate that laser fluence strongly affects the structural ordering, defect density, and optical properties of SnS–PVA system. Furthermore, similar photoluminescence properties in the 600–900 nm range have been reported.^40,41^ These properties are attributed to transformations associated with defects or impurities in the SnS films.

Fig. 3(d) shows the Raman spectra of tin sulfide nanoparticles synthesized at different laser fluences, recorded in the 65–300 cm^−1^ spectral range. SnS has an orthorhombic crystal structure with eight atoms per unit cell. For this orthorhombic structure, the 24 vibrational modes are represented by the following irreducible representations at the center of the Brillouin zone:^42^*Γ* = 4A_g_ + 2B_1g_ + 4B_2g_ + 2B_3g_ + 2A_u_ + 4B_1u_ + 2B_2u_ + 4B_3u_

SnS exhibits 21 optical vibrational modes, of which 12 are Raman-active modes (4 A_g_ modes, 2 B_1g_ modes, 4 B_2g_ modes, and 2 B_3g_ modes), and the remaining modes are either infrared-active or silent (A_u_ modes).^36^ As shown in Fig. 3(d), Raman spectroscopy results revealed prominent Raman-active vibrational modes at ∼68, 95, 108, 164, 180, 190, 218, 232, and 292 cm^−1^. As indicated in previous reports, their appearance is attributed to Sn–S bond vibrations.^43–46^ The peaks at 68, 164, and 292 cm^−1^ are assigned to the B_1g_, B_3g_ and B_2g_ modes, respectively, and the peaks at 95, 108, 190, 218, and 232 cm^−1^ are attributed to the A_g_ mode. Furthermore, as can be seen in the Fig. 3(d), the peaks of the A_g_ and B_3g_ modes are slight shift toward higher wavenumbers is observed for some Raman modes compared to bulk SnS, while others remain nearly unchanged. The assignment of Raman-active modes and their comparison to single-crystal SnS and relevant literature values are clearly summarized in Table 4. These shifts in peaks are due to the small particle size (quantum confinement) and lattice microstrain, which makes phonon modes highly sensitive to structural perturbations and lattice disorder. This finding is reflected in the phonon spectra through the shifting and broadening of peak positions.^43^ The obtained results indicate that the decrease in the Raman peak intensity may be attributed to enhanced surface-related effects as well as the phonon confinement due to reduced crystallinity and increased structural disorder in the synthesized nanoparticles. This, in turn, leads to reduced Raman scattering intensity. The observed Raman modes are characteristic of orthorhombic SnS, confirming successful formation of the SnS phase within the SnS–PVA nanocomposite.

**Table 4:** Comparison of the obtained Raman peak positions with the reported Raman modes of orthorhombic SnS

Observed Raman peak (cm^−1^)	SnS single crystal (cm^−1^)	Vibrational mode	Reference
68	70	B_1g_	45 and 48
96	95	A_g_	43–45
108	111	A_g_	44
164	164	B_3g_	43,44,49
180	180–182	A_g_	45
191	191–194	A_g_-dominated mode	43 and 44
218	218	Ag	43 and 50
232	238	A_g_	43 and 46
292	290	B_2g_	43,45,51

The improved Raman intensity and reduced peak broadening of the sample prepared at 45 J cm^−2^ indicate improvement of crystalline quality.^46,47^ As the laser fluence increased, a gradual decrease in the intensity of the Raman peaks and slight broadening were detected, possibly due to increased structural disorder and defect formation. The intensity and peak broadening as a function of the laser fluence indicate structural disorder, size distribution, and crystal defects, which are in good agreement with the results obtained from field emission scanning electron microscopy (FESEM). These Raman results indicate that the laser fluence may directly affect the structural ordering, crystallinity, and the nature of the interaction between the nanoparticles and the PVA matrix in the SnS–PVA system, which in turn affects the optical and electronic properties of the material.

**Fig. 4:**
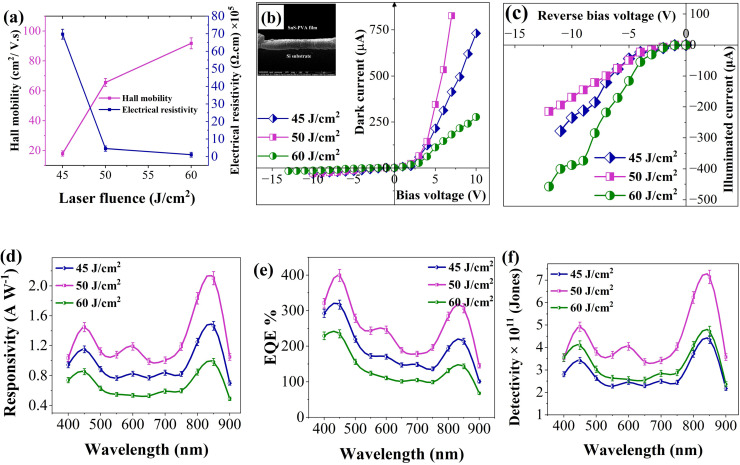
(a) Charge carrier concentration, Hall mobility, and electrical resistivity of SnS films as functions of laser fluence, (b) dark *I*–*V* characteristics, (c) photo *I*–*V* curves for photodetectors prepared at different laser fluences 45, 50, and 60 J cm^−2^, (d) spectral responsivity (*R*_*λ*_) (e) external quantum efficiency (EQE), (f) specific detectivity (D*). error bars in panels (d)–(f) represent the standard deviation of three independent measurements (*n* = 3).

Hall effect measurements showed that the SnS nanostructure films have a positive Hall coefficient, indicating these films are p-type. Laser fluence influences defect states and consequently affects the electrical properties of the film. The Hall mobility and electrical resistivity of SnS nanostructure films as a function of laser fluence are depicted in Fig. 4(a). As shown, the hole mobility increases from 18 to 91.8 cm^2^ V^−1^ s^−1^ as laser fluence increases from 45 to 60 J cm^−2^, which may be attributed to improved electrical pathways resulting from the morphological evolution of the SnS nanostructures. The electrical resistivity of SnS film was found to decrease from 69.74 × 10^5^ Ω cm at 45 J cm^−2^ to 1.095 × 10^5^ Ω cm with increasing laser fluence. The electrical resistivity decreased with increase in Hall mobility indicating that the SnS film had more efficient charge transport at higher laser fluence. The film prepared at 50 J cm^−2^ had the highest carrier concentration in agreement with its superior optoelectronic performance. In contrast, carrier concentration decreased at 60 J cm^−2^, which may be attributed to the formation of structural defects or increased surface roughness, acting as trapping and recombination centers, thereby reducing the electrically active carrier density.

Despite the decrease in crystallite size and the increase in dislocation density, Hall mobility continued to increase with laser fluence. However, carrier mobility alone does not determine photodetector performance. The sample prepared at 50 J cm^−2^ exhibited the optimum combination of relatively high carrier mobility (65.6 cm^2^ V^−1^ s^−1^), the highest carrier concentration (2.74 × 10^11^ cm^−3^), and lower structural disorder than the sample prepared at 60 J cm^−2^. In contrast, although the Hall mobility further increased to 91.8 cm^2^ V^−1^ s^−1^ at 60 J cm^−2^, the significant reduction in carrier concentration, together with the increase in lattice defects and microstrain, indicates enhanced carrier trapping and recombination. Therefore, the overall optoelectronic performance is governed by the balance among carrier mobility, carrier concentration, and defect density rather than by carrier mobility alone. This behavior is most likely related to the film's morphological evolution, which provided more continuous transport pathways and enhanced connectivity between nearby nanostructures. Accordingly, the improved transport pathways may have compensated, to some extent, for the increase in structural defects Fig. 4(b) shows the current–voltage (*I*–*V*) characteristics in the dark of the p-SnS/n-Si heterojunction fabricated with different laser fluences in both the forward and reverse directions within a voltage range of −13 to 13 V. The inset shows the cross-sectional FESEM image of the SnS/Si interface prepared at 50 J cm^−2^, revealing an average SnS film thickness of approximately 515 nm. Fig. 4(b) displays that the p-SnS/n-Si heterojunctions exhibit rectifying properties. An exponential increase in the forward current with the forward bias voltage occurs because the bias voltage reduces the potential barriers and facilitates charge carrier transport across the interface by reducing the effective barrier height. Conversely, the reverse bias current increases with the voltage very slowly. The heterojunction prepared at 50 J cm^−2^ exhibits the highest forward rectifying current compared to the other samples. This can be attributed to the higher carrier concentration and the low electrical resistivity of the SnS layer prepared at 50 J cm^−2^ (Table 5).

**Table 5:** Comparison of electrical parameters obtained from the Hall effect measurement of SnS–PVA nanostructures

Laser fluence (J cm^−2^)	Carrier concentration (cm^−3^)	Hall mobility (cm^2^ V^−1^ s^−1^)	Electrical resistivity (Ω cm) × 10^5^
45	4.919 × 10^10^	18	69.74
50	2.74 × 10^11^	65.6	4.587
60	6.202 × 10^10^	91.8	1.095

Two regions were found to comprise the forward current, the first region, referred to as the recombination current, takes place at a low bias voltage. The depletion region stays large at these low voltages because there is not enough applied voltage to significantly lower the barrier height. At higher forward bias voltages, the diffusion current predominates in the second region.

The photo *I*–*V* characteristics of SnS/Si heterojunction photodetectors at different light intensities are shown in Fig. 4(c). As the photodetector is illuminated (SnS-side) with white light, e–h pairs are produced in the depletion region, and the internal electric field efficiently separates the photogenerated electron–hole pairs, thereby suppressing carrier recombination and enhancing charge collection. The highest photocurrent was found for the photodetector fabricated at 60 J cm^−2^, which may be attributed to its higher carrier mobility and lower electrical resistivity, facilitating efficient transport and collection of photogenerated carriers. Furthermore, the photocurrent increases with increasing light intensity owing to the generation of a larger number of electron–hole pairs.

The photodetectors' linearity properties are demonstrated by the lack of saturation in the photocurrent at high light intensities. Furthermore, a constant rise in the obtained photocurrent indicates a trap-assisted photoconductive gain; specifically, the trap states that arise permit extended carrier lifetime, which enhances the photocurrent collection phenomenon.

As illustrated in Fig. 4(d), the spectral response of SnS/n-Si heterojunction photodetectors over the wavelength range of 400 to 900 nm. These photodetectors were fabricated at different laser fluences at a bias voltage of −10 V. Responsivity (*R*_*λ*_)is an important parameter to evaluate the performance of the photodetector. The spectral responsivity (*R*_*λ*_) was calculated from the ratio of the photocurrent to the incident optical power according to:^4^8
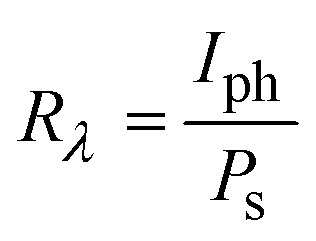


The SnS photodetector fabricated at 50 J cm^−2^ exhibited two pronounced responsivity peaks, the first near 450 nm in the visible light range and the second at 850 nm. The first peak is due to the extension of the depletion region towards the SnS nanoparticles layer because of the difference in the carrier concentration in the two materials. The secondary peak is attributed to the absorption edge of silicon substrate. Moreover, the results show a significant enhancement of responsivity at the higher wavelengths, which is attributed to the enhancement of the photocarrier generation inside the silicon substrate. Besides, the application of PVA helps to reduce the aggregation of the nanoparticles, optimize the charge carrier transport pathways, and enhance the light trapping efficiency.^52–54^

The sample prepared at 50 J cm^−2^ showed the highest responsivity 1.45 A W^−1^ at 450 nm followed by a weaker response at 600 nm, 1.19 A W^−1^. The photodetectors fabricated at 45 and 60 J cm^−2^ peaks revealed a predominant peak at 450 nm, with 1.15 and 0.85 A W^−1^, respectively.

However, when the wavelength is increased from 850 to 900 nm (towards the near infrared region), the responsivity gradually decreases because of the lower absorption coefficient of the SnS–PVA layer at longer wavelengths, which reduces the efficiency of electron–hole pair generation and carrier collection.^40^ This behavior reduces the probability of photogenerated carrier collection, resulting in lower responsivity at longer wavelengths.

The high response around 450 nm is attributed to the optical transitions related to the absorption edge of the SnS layer. In additional, surface defects, or quantum confinement in the SnS nanoparticles may cause the broadening of the absorption spectrum into the visible regions, which may lead to a higher response at shorter wavelengths.

The different responses to the various laser fluence levels are explained by the different particle sizes, surface structures, and defect densities of the nanoparticles generated by laser ablation. The sample prepared at 50 J cm^−2^ exhibited the highest optical responsivity of all the prepared samples, indicating that this fluence represents the best balance between structural ordering, nanoparticle size distribution, and charge carrier transport efficiency.^55,56^ The lower response of the sample fabricated at 60 J cm^−2^ can be explained by considering both the Hall effect and structural analyses. Although Hall mobility increased to 91.8 cm^2^ V^−1^ s^−1^, the carrier concentration decreased markedly from 2.74 × 10^11^ cm^−3^ at 50 J cm^−2^ to 6.20 × 10^10^ cm^−3^ at 60 J cm^−2^. In contrast, the XRD results indicated an increase in microstrain and dislocation density, indicating a higher density of structural defects. These defects serve as carrier trapping and recombination centers and reduce number of the photogenerated carriers that can reach the electrodes. Consequently, the positive effect of the higher mobility is overshadowed by the decrease in carrier concentration and the increase in recombination leading to a decrease in responsivity and the photodetection efficiency.

Fig. 4(d) shows the external quantum efficiency EQE of the p-SnS/n-Si photodetector fabricated at different laser fluences. The photodetectors fabricated at 45, 50, and 60 J cm^−2^ have EQE of 317%, 399%, and 235%, respectively, at wavelength 450 nm. The maximum EQE at 450 nm can be attributed to the absorption edge of the SnS layer.^57^ A secondary enhancement in EQE at 850 nm is associated with the increased contribution of the silicon substrate to photocarrier generation in the near-infrared region. A high absorption coefficient of the layer leads to a very small absorption depth, so absorption takes place on the surface of the layer and enhances responsivity at short wavelengths. The presence of an internal gain mechanism within the p-SnS/n-Si heterojunction is the main reason for reaching the EQE > 100%. This behavior is explained by carrier trapping at the SnS/Si interface and defect-related states in the SnS layer, which lengthens the carrier lifetime relative to transit time and produces photoconductive gain. These EQE values are comparable to those of previous studies.^58–62^

**Fig. 5:**
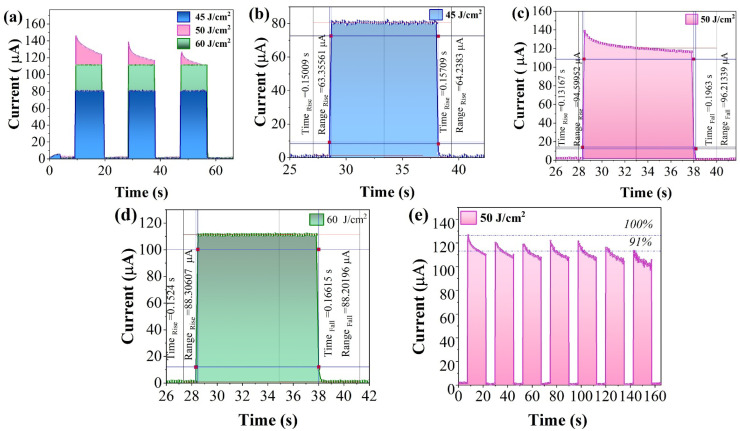
(a) *I*–*t* switching characteristics of a p-SnS–PVA/n-Si photodetector at different laser fluences (b) *I*–*t* of the photodetector fabricated at 45 J cm^−2^, (c) *I*–*t* of the photodetector fabricated at 50 J cm^−2^, and (d) *I*–*t* of the photodetector fabricated at 60 J cm^−2^, (e) long-term stability of the p-SnS–PVA/n-Si photodetector fabricated at a laser fluence of 50 J cm^−2^ under repeated ON/OFF illumination cycles over 7 days.

The photodetector fabricated at 50 J cm^−2^ showed the highest EQE, which suggested the optimum balance between the structural ordering and defect states. This observation is also consistent with the Hall effect measurements, which showed a significant decrease in carrier concentration at 60 J cm^−2^ despite the increase in Hall mobility. The reduced carrier concentration, together with increased defect-assisted recombination, limits the collection efficiency of photogenerated carriers and consequently lowers the EQE. This improvement may be associated with more efficient carrier collection and reduced carrier recombination, which normally play a crucial role in the improvement of collection efficiency of the photogenerated charge carriers. In contrast, the photodetector prepared at 60 J cm^−2^ exhibited the lowest EQE, which may be attributed to defect-related recombination that reduces the collection efficiency of photogenerated carriers.^58,59^ The specific detectivity of the photodetector can be calculated using the following relationship:9
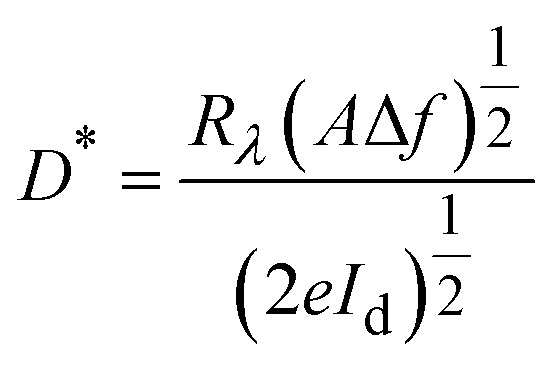
where *I*_d_ is the dark current, Δ*f* is the bandwidth, *e* is the electron charge and *A* is the photosensitive area of the photodetector. The specific detectivity (D*) of the photodetector fabricated at 50 J cm^−2^ is higher than that fabricated at 45 and 60 J cm^−2^, as shown in Fig. 4(e). The photodetector fabricated at 50 J cm^−2^ has D* = 4.9 × 10^11^ Jones at 450 nm and the corresponding noise equivalent power (NEP) = 2.04 p W. The photodetectors fabricated at 45 and 60 J cm^−2^, in comparison, had lower D*. The fabricated photodetectors' performance is comparable to those of other heterojunction silicon photodetectors, as presented in Table 6. The decrease in the D* may be attributed to increases the surface leakage current arise from the defects and surface states, which will reflect the photodetector's ability to detect weak optical signal. Fig. 5(a) shows the dynamic photoresponse of the SnS/Si photodetectors as a function of the laser fluence. The measurements were performed over a 65 s interval. As illustrated in the Fig. 5(c), the photodetector fabricated at a laser fluence of 50 J cm^−2^ showed the fastest rise and decay times with ratio *τ*_r_/*τ*_f_ = 0.13 s/0.12 s. This behavior can probably be attributed to optimized structural characteristics and lower defect density of the SnS layer prepared at this laser fluence. The sample prepared at 45 J cm^−2^ showed the lowest response with *τ*_r_/*τ*_f_ = 0.15 s/0.16 s, which can be attributed to less efficient charge transfer as shown in Fig. 5(b). On the contrary, sample 60 J cm^−2^ exhibited a *τ*_r_/*τ*_f_ value of 0.152 s/0.17 s with stable and repeatable photoresponse, indicating the enhanced signal stability as shown in Fig. 5(d). The slight decrease in photocurrent compared with the sample prepared at 50 J cm^−2^ may be associated with increased carrier trapping caused by surface defects generated at higher laser fluence. Optimizing the laser fluence during film deposition improves the photodetector's sensitivity and response speed. Table 6 summarizes the figures of merit of the photodetectors fabricated at various laser fluences.

**Table 6:** Influence of laser fluence on the figures of merit of p-SnS/n-Si photodetector at 450 nm

Laser fluence (J cm^−2^)	*R*_*λ*_ (A W^−1^)	EQE (%)	D* (Jones)×10^11^	NEP (pW)	Rise time (s)	Decay time (s)
45	1.15	317	3.4	2.9	0.15	0.16
50	1.45	399	4.9	2.04	0.13	0.12
60	0.85	235	4.09	2.4	0.152	0.17

The long-term stability test for p-SnS/n-Si photodetector prepared at a laser fluence of 50 J cm^−2^, is demonstrated in Fig. 5(e). The stability of the photodetector was evaluated over 7 days by seven daily automatic ON/OFF cycles (30 s each) and a 20 s pulse width. The optical device operated continuously without interruption, demonstrating stable photodetector performance. However, a slight alteration in the value of the photocurrent was detected over 5 days, where the fabricated photodetector degraded by about 9% from its initial photocurrent value by the end of the 7 days period. The behavior indicates the efficient charge transport and collection processes and the stability of the structure of the SnS/n-Si heterojunction during continuous operation. These results demonstrate good operational stability, indicating demonstrate good long-term operational stability. These outstanding performances could be potentially applicable for practical optoelectronic applications.

The obtained results indicate that the photodetector performance is strongly dependent on the structural characteristics of the synthesized SnS nanoparticles, which are governed by the applied laser fluence. At a laser fluence of 50 J cm^−2^, the SnS nanoparticles exhibited improved crystallinity together with a partially overlapping cauliflower-like morphology. These structural features enhanced light absorption and promoted more efficient separation and transport of photogenerated charge carriers across the p-SnS/n-Si heterojunction. Thus, the device fabricated at this fluence showed the maximum values of photocurrent, responsivity, detectivity, and external quantum efficiency. In contrast, increasing the laser fluence to 60 J cm^−2^ led to the formation of nanorod-like structures with an increased optical energy gap, which deteriorated the photoresponse of the device. These results suggest that the structural evolution of SnS nanoparticles induced by laser fluence is an important factor in determining their optoelectronic properties and photodetector performance. Table 7 compares the performance of the present SnS–PVA/Si photodetector with previously reported SnS-based photodetectors prepared using different fabrication techniques. Although some devices exhibit higher responsivity or detectivity, they generally require more complex fabrication processes or hybrid device architectures. In contrast, the present device was fabricated by a simple PLAL route and exhibits a competitive responsivity of 1.45 A W^−1^, a detectivity of 4.9 × 10^11^ Jones, an EQE of 399%, and fast response/recovery times of 0.13/0.12 s. These results demonstrate that the proposed fabrication approach offers an effective balance between device performance and process simplicity.

**Table 7:** Comparison of the obtained figures of merit with previously published works

Photodetector type	Preparation method	Peak wavelength (nm)	Responsivity *R*_*λ*_ (A W^−1^)	Detectivity (Jones)	External quantum efficiency%	*τ*_r_/*τ*_f_	Reference
SnS thin film	Thermal evaporation	532	0.06	6.8 × 10^10^	14	4.8/5.2	60
SnS thin film	PLAL + Spin-coating	Up to 980	10^−7^	10^6^	8.48 × 10^6^	1.8/4.6	61
SnS/SnSe_2_	Two-step physical vapor epitaxial growth	420	4.76 × 10^−6^	___	___	0.0296/0.1094	62
SnS	Two-step physical vapor epitaxial growth	UV-visible	1.09 × 10^−6^	____	___	___	62
SnS–graphene hybrid	PLIL + spin-coating	UV-Vis-NIR (200–980)	10^−5^	10^7^	___	1.1	22
SnS–Si hybrid	PLAL + pulsed laser fragmentation	Up to 1064	10^−9^–10^−7^	10^5^–10^6^	___	___	63
SnS/Si	Chemical bath deposition (CBD)	636	68.21	6.87 ×10^13^	1.32 × 10^4^	0.194/0.941	64
SnS QDs/MAPbI3	Spin-coating	500–750	0.32	1.9 × 10^12^	80	0.047/0.043	65
Hetero SnS/SnS_2_	Laser irradiation (CW)	532	4	___	___	0.0018/0.016	23
SnS–PVA/Si	PLAL	450	1.45	4.9 × 10^11^	399%	0.13/0.12	This work

## 4 Conclusion

We successfully fabricated a low-cost, high-performance p-SnS/n-Si heterojunction photodetector by depositing a nanostructured SnS film on a silicon substrate prepared *via* laser ablation of a tin target in a PVA–Na_2_S liquid medium at different laser fluences. The variation in the optical energy gap was closely related to the structural and morphological changes induced by the applied laser fluence. The electrical and optoelectronic characteristics of SnS/Si heterojunctions exhibited significant diode behavior with evident rectification. The photodetector prepared at 50 J cm^−2^ exhibited the best photodetection performance. It achieved a maximum responsivity of 1.45 A W^−1^, a maximum EQE of 399%, and the highest detectivity of 4.9 ×10^11^ Jones at 450 nm. The fabricated photodetector showed the fastest rise and decay times of 0.13 s and 0.12 s, respectively, at a laser fluence of 50 J cm^−2^. The results demonstrate that laser fluence is a key processing parameter for tailoring the structural, morphological, and optical properties of SnS nanoparticles, which directly influence the photodetection performance of the fabricated SnS/Si heterojunction. The proposed PLAL approach provides a promising route for the fabrication of low-cost, high-performance silicon heterojunction photodetectors by controlling the laser fluence. The developed SnS/n-Si photodetector exhibits competitive photodetection performance over a broad visible-to-near-infrared spectral range. These advantages render it an attractive candidate for future low-cost optoelectronic and photonic technologies.

## Author contributions

Fatima M. Abdulaziz: conceptualization, methodology, investigation, formal analysis, data curation, visualization. Raid A. Ismail: conceptualization, methodology, supervision, interpretation of results, writing – review and editing. Alwan M. Alwan: formal analysis, visualization, supervision, interpretation of results, writing – review and editing. All authors discussed the results, contributed to the final form of the manuscript, and approved the submitted version.

## Conflicts of interest

The authors declare that they have no conflicts of interests.

## Data Availability

The datasets generated during the current study are available from the corresponding authors on reasonable request.
